# zDHHC9 and the Control of Natriuretic Peptide Secretion by the Heart∗

**DOI:** 10.1016/j.jacbts.2023.02.013

**Published:** 2023-05-22

**Authors:** William Fuller

**Affiliations:** School of Cardiovascular & Metabolic Health, University of Glasgow, Glasgow, United Kingdom

**Keywords:** acylation, heart failure, atrial natriuretic peptide, brain natriuretic peptide, palmitoylation

**Figure:**
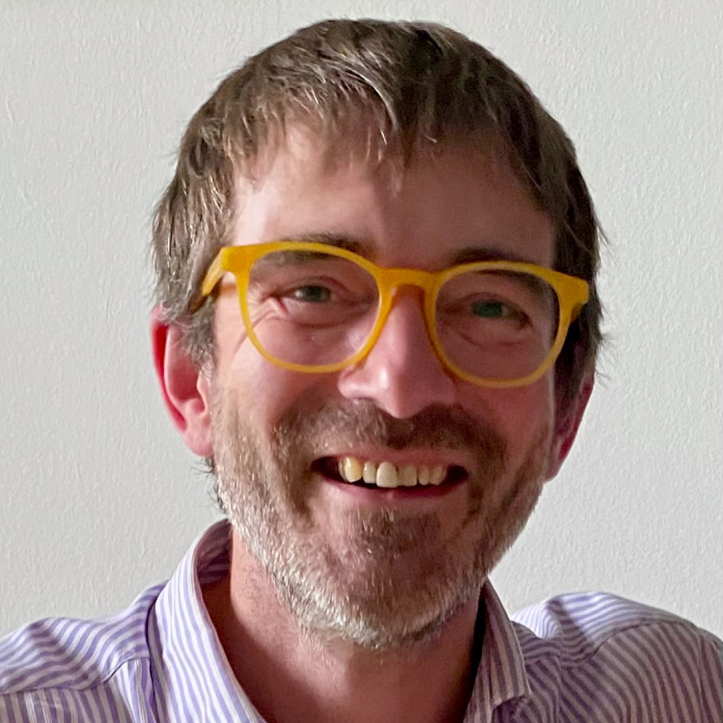

Over the last decade, the therapeutic tractability of targeting the natriuretic peptide family (atrial natriuretic peptide [ANP] and brain natriuretic peptide) has been explored extensively. This family of secreted signaling molecules mediates numerous beneficial effects in a setting of heart failure (HF). For example, the vasodilation, natriuresis, and diuresis evoked by natriuretic peptides dampen the renin-angiotensin-aldosterone system, decreasing the cardiac workload, and protecting the heart from hypertrophy and fibrosis. The administration of recombinant ANP itself (carperitide) is not widely adopted in the clinic. In contrast, blocking natriuretic peptide breakdown using sacubitril to inhibit the membrane bound endopeptidase neprilysin showed spectacular success (in combination with an angiotensin receptor blocker) in Paradigm-HF (Prospective Comparison of ARNI [Angiotensin Receptor–Neprilysin Inhibitor] With ACEI [Angiotensin-Converting–Enzyme Inhibitor] to Determine Impact on Global Mortality and Morbidity in Heart Failure). This led to the adoption of sacubitril/valsartan as a first-line treatment for HF with reduced ejection fraction.^1^

Given these significant benefits of enhancing ANP and brain natriuretic peptide bioavailability for patients with HF, remarkably little is known about the mechanisms controlling their production and secretion by the heart. In this issue of *JACC: Basic to Translational Science*, Essandoh et al^2^ provide important new insights into these mechanisms that offer the potential to open new therapeutic avenues for HF.

## New Insight: Post-translational Control of ANP Secretion

In the field of the post-translational regulation of protein behavior, reversible protein phosphorylation has long been king. The pathogenesis of HF is linked intimately with aberrant phosphorylation of contractile and structural myofilament proteins, ion transporters, transcription factors, histone deacetylases, and many others. However, phosphorylation is just one of >400 chemically distinct post-translational modifications of proteins that have been described to date. As the search for new therapeutic targets for HF expands, it is undoubtedly important to expand our horizons beyond only protein phosphorylation.

S-palmitoylation, S-acylation, or simply ‘palmitoylation’ describes the reversible attachment of a saturated fatty acid, usually 16-carbon palmitic acid, to protein cysteines through a thioester bond. Catalyzed by integral membrane zinc finger DHHC-domain containing palmitoyl acyl transferases (zDHHC-PATs) and reversed by thioesterases, palmitoylation occurs dynamically throughout the cell, controlling an ever-expanding repertoire of physiological and pathophysiological events.^3^ Typically when a substrate protein is palmitoylated, the requirement for the fatty acid that has been conjugated to this protein to reside in a hydrophobic environment means that this protein becomes anchored to a membrane. The fact that different members of the zDHHC-PAT family are located throughout the secretory pathway means that many different protein families can be anchored reversibly to many different cellular compartments. Indeed, >10% of the human proteome is probably modified in this way.

Studies that have characterized reversible palmitoylation in the heart to date have focused principally on the regulation of integral membrane ion transporters and the impact of dynamic palmitoylation at the cell surface on electrical and contractile behavior of cardiac myocytes.^3^ Changes in expression and activity of the cellular palmitoylation machinery and the contribution of aberrant substrate palmitoylation to HF have only recently started to be dissected.^4^ Essandoh et al^2^ found that the cardiac-specific overexpression of the Golgi-localized enzyme zDHHC9 caused lethal dilated cardiomyopathy accompanied by profound enlargement of both atria and ventricles. zDHHC9 is an important enzyme outside the heart because it palmitoylates small G proteins, including Ras isoforms, and hence controls signal transduction and cell growth, proliferation, and migration. Interestingly, quantitative proteomics found that one of the biggest changes in palmitoylation induced by zDHHC9 overexpression in the heart was in the protein Rab3gap1, not a small G protein itself, but an activator of GTPase activity for the small G protein Rab3. Having validated Rab3gap1 as a zDHHC9 substrate, Essandoh et al^2^ went on to demonstrate that, in their zDHHC9 overexpressing hearts, Rab3gap1 is trapped at the Golgi. Simply put, palmitoylation seems to sequester Rab3gap1 away from its usual partner Rab3a, although dynamic imaging of Rab3gap1 localization is really needed to tease out the subtleties here. In the absence of its GTPase-activating partner, Rab3a becomes trapped in a GTP-loaded state, unable to mediate fusion of secretory vesicles with the myocyte plasma membrane. These are the very secretory vesicles loaded with ANP, and Essandoh et al^2^ demonstrate that ANP secretion from both cultured myocytes and the heart in vivo is impaired when zDHHC9 is overexpressed. The converse was also found to be true: acute silencing of zDHHC9 enhanced ANP secretion induced by phenylephrine treatment of cultured myocytes, and basal ANP secretion was enhanced when Rab3gap1 (but not an inactive mutant) was overexpressed.

These new findings position the zDHHC9/Rab3gap1/Rab3a axis centrally in the control of natriuretic peptide secretion by the heart. At the heart of this relationship, zDHHC9 activity seems to be the most important factor, because this sequesters Rab3gap1 at the Golgi and can consequently abrogate Rab3a-mediated vesicle fusion and ANP secretion. Interestingly, ANP secretion induced by phenylephrine is associated with enhanced Rab3gap1 palmitoylation, a sign that perhaps zDHHC9 activity is increased to limit ANP secretion in a classic negative feedback loop. Strategies to promote nucleotide cycling on Rab3a, globally decrease zDHHC9 activity, or better still specifically decrease zDHHC9 recruitment and palmitoylation of Rab3gap1, may consequently offer promise for HF because they will increase circulating concentrations of natriuretic peptides—with universally accepted beneficial consequences.

## New Questions: Cellular Mechanisms Regulating zDHHC9 and Rab3gap1

The transcriptomic signature of HF with reduced ejection fraction suggests a modest decrease in zDHHC9 expression, and no significant remodeling of Rab3gap1 or Rab3a.^5^ However, the contribution of abnormal Rab3gap1 palmitoylation, localization, or activity to human HF remains to be established. Central to this line of inquiry will be the activity, rather than simply the expression, of zDHHC9. Despite the importance of palmitoylation as a post-translational regulatory mechanism, remarkably little is known about the cellular mechanisms regulating zDHHC-PAT activity. Some members of this family, including zDHHC9 and zDHHC5, are proposed to require an accessory protein to regulate their activity, stability, or localization. However, Essandoh et al^2^ find that the classic zDHHC9 partner protein GCP-16 seems to have no role in controlling cardiac ANP secretion. Unlike protein kinases, few signaling pathways have been established to directly regulate zDHHC-PAT enzymatic activity. Instead, regulation frequently occurs via an effect on the enzyme's ability to recognize and palmitoylate its substrates.^3^ Given the broad importance of zDHHC9 substrates for numerous cellular events, understanding cellular control of its expression and activity is an important future priority, especially because the profound cardiomyopathy phenotype caused by cardiac-specific zDHHC9 overexpression is unlikely to be attributable solely to impaired ANP secretion. There may be other zDHHC9 substrates still to be identified whose palmitoylation contributes to the development of HF.

For every palmitoylated substrate, in addition to a palmitoylating enzyme there is also an erasing, depalmitoylating enzyme to consider. The thioesterase family, almost entirely composed of serine hydrolase enzymes, is rather less well-characterized than the zDHHC-PATs. Given the importance of Rab3gap1 palmitoylation, identifying the enzyme that depalmitoylates it (and how this is regulated), whose activity likely enhances ANP secretion, is also a high priority. Ultimately, the findings from Essandoh et al^2^ mean that any intervention that can unlock Rab3gap1 from its confinement in the Golgi offers therapeutic promise for HF.

## Funding Support and Author Disclosures

The author acknowledges financial support from the British Heart Foundation, grant reference PG/22/10847.
